# Sexual and Reproductive Health Services Utilization among Wolaita Sodo University Students, Ethiopia: A Mixed Method Approach

**DOI:** 10.1155/2021/2415023

**Published:** 2021-12-17

**Authors:** Muluken Gunta, Temesgen Tantu, Sintayehu Wolka, Mengistu Meskele, Asaminew Ayza, Bereket Duko

**Affiliations:** ^1^Wolaita Zone Health Department, Wolaita Sodo, Ethiopia; ^2^Department of Obstetrics and Gynecology, Wolkite University, Wolkite, Ethiopia; ^3^Health System Strengthening Special Support Directorate, Federal Ministry of Health, Addis Ababa, Ethiopia; ^4^School of Public Health, College of Health Science and Medicine, Wolaita Sodo University, Wolaita Sodo, Ethiopia; ^5^Faculity of Health Sciences, College of Medicine and Health Sciences, Hawassa University, Hawassa, Ethiopia; ^6^Curtin School of Population Health, Curtin University, Perth, Australia

## Abstract

**Background:**

Youths have been facing different sexual and reproductive health problems such as HIV infections and unplanned pregnancies. Therefore, this study aimed to assess reproductive health services utilization and their associated factors among Wolaita Sodo University students in Wolaita Sodo, Ethiopia.

**Methods:**

We conducted an institutionally-based mixed-method study among 759 regular undergraduate university students. Multistage random sampling and purposive sampling techniques have been used to recruit students for the quantitative and qualitative studies, respectively. A pretested self-administered questionnaire was used to collect the data. A logistic regression model was used for quantitative data analysis, whereas thematic analysis was used for qualitative data. We used open-code software-assisted qualitative data analysis. The statistical significance was declared at a *P* value less than 0.05.

**Results:**

We found that 378 (49.8%) (95% CI: 46.20–53.34) of respondents had utilized sexual and reproductive health services within the 12 months preceding the current survey. Being a first-year student (AOR = 1.57, 95% CI: 1.01–2.46), having ever had sexual intercourse (AOR = 5.12, 95% CI: 3.31, 7.96), participating in peer-to-peer discussion (AOR = 1.46, 95% CI: 1.02–2.02), and having ever had sexual transmitted infection syndrome (AOR = 3.91, 95% CI: 1.41–10.85) have increased the odds of using sexual and reproductive health services.

**Conclusion:**

Sexual and reproductive health services utilization among university students was inadequate and affected by several factors. Therefore, strengthening peer support networks and addressing the gap in services were highly recommended.

## 1. Background

Sexual and reproductive health is a state of complete physical, mental, and social well-being in all matters related to the reproductive system at all stages of life [1, 2]. The United Nations International Conference on Population and Development (UNICPD) has grouped reproductive health within a broader sociocultural context that includes gender roles, respect, and protection of human rights [1].

Young people aged 15–24 experienced the highest rates of sexually transmitted infections of any age group in 2015 globally [2]. They also experience high rates of early and unintended pregnancy, unsafe abortion, and sexually transmitted infections including HIV [3]. Most importantly, the problem is becoming prevalent among youth in higher academic institutions [4, 5]. It has been reported that students in higher academic institutions are more likely to be exposed to a range of risky sexual behaviours [6].

Estimates from the 2016 population demography of Ethiopia suggested that 33.8% of the population was in the age range of 10–24 years [7]. Although large numbers of youths in this age range are attending different universities within the country, significant numbers did not reach a full range of sexual and reproductive health services yet [4], indicating students have less access to information, services, and resources [8]. Thus, university students in Ethiopia often had low utilization of sexual and reproductive health services compared to the other groups of the population [9, 10].

To our knowledge, little has been investigated about sexual and reproductive health services utilization and their correlates in the context of Ethiopian higher education in general and Wolaita Sodo University in particular. Therefore, this study aimed to assess the prevalence of sexual and reproductive health service utilization and its associated factors among regular students attending Wolaita Sodo University, Ethiopia.

## 2. Methods

### 2.1. Study Setting and Design

An institutionally-based mixed-methods research approach was followed to conduct this study among Wolaita Sodo University students. Wolaita Sodo University is one of the second generation universities in Ethiopia, located 315 km away from Addis Ababa, the capital of Ethiopia.

### 2.2. Sample Size and Sampling Techniques

**Figure 1:**
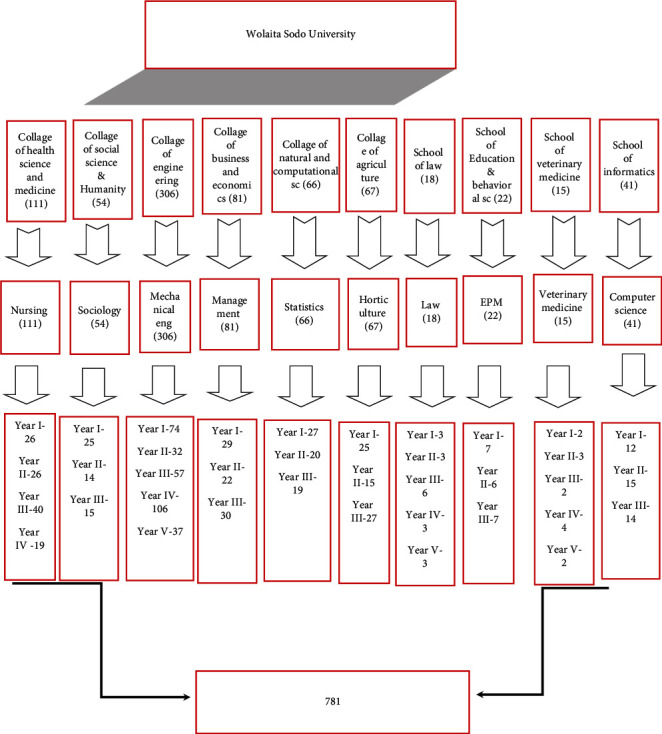
Schematic presentation of the sampling procedure to select study participants at WSU.

The sample size for this study was calculated by using a single population proportion formula with the assumption of a 95% confidence level, the margin of error of 5%, and the prevalence of youth-friendly reproductive health service utilization (63.8%), taken from a study of Harar, Ethiopia [11]. After considering a 10% nonresponse rate, the total sample size was estimated to be 781. Multistage random sampling and purposive sampling techniques have been used to recruit students for the quantitative and qualitative studies, respectively (Figure 1). The university had 12,092 regular undergraduate students in its six colleges and four schools. From each college and school, one department was selected by a lottery method. The proportional allocation method was used to choose study participants from each department. One class from each year of education was selected by a lottery method. A total of 37 classes were selected from randomly selected ten departments. Study participants were selected from the classes by using simple random sampling methods. For the qualitative study, the purposive sampling technique was employed to identify the list of potential in-depth interview participants. A total of eleven key informant interviews were conducted with selected SRH service providers and students who were not included in the quantitative survey. Numbers of interviewees were sought out until idea saturation was reached.

### 2.3. Data Collection

A structured and self-administrated questionnaire was used for quantitative data collection. Data was collected by eight trained data collectors. Supervisors assigned students to classrooms and oriented students on how collected data will be handled. Questionnaires were stored in baskets which were prepared for the study purpose. The data collection process was closely supervised. A semistructured interview guide, notetaking, and tape recording were used to gather qualitative data. A trained data collector made the recording of audio data in quite a site with notetaking.

### 2.4. Data Processing and Analysis

We entered and cleaned the quantitative data by using EpiData version 3.1 and exported it to SPSS version 21 for analysis. We used descriptive statistics to calculate the frequencies and percentages of different variables. We used bivariate and multivariable logistic regression analyses along with the odds ratio and 95% confidence intervals. Multivariable analysis was used to adjust for possible confounders and come up with significant predictors. The level of significance was declared at a *p* value of less than 0.05. In the qualitative part, the information recorded during data collection was first transcribed into hard copy documents. The transcription and notes taken in the field were translated into English. Coding and categorizing were done using OpenCode version 3.6 software. Finally, qualitative data were analyzed thematically.

### 2.5. Measurements

The knowledge score was computed based on four awareness questions (with “Yes”/”No” answers). Those who responded 0–2 correct answers were categorized as “Not knowledgeable,” while those who responded 3-4 correct answers were classified as “Knowledgeable.” Attitude questions were measured using a Likert scale. But, to analyze the outcome variable, the composite variable was dichotomized using the median of the ten attitude questions. Those who scored less than or equal to the median score were categorized as having “Negative attitude.” In contrast, those who scored more than the median score on attitude questions were classified as having “Positive attitude.”

### 2.6. Operational Definition

SRH service utilization includes taking at least one of the following services like SRH counselling, condoms, abortion care, VCT, and family planning or STI treatment delivered at health facilities within the past 12 months (one year).

## 3. Results

A total of 759 students participated in this study, which made the response rate 97.2%. Of 759 respondents, 411 (54.2%) were males and 348 (45.8%) were females. The median age of respondents was 2 1(IQR = 2). Regarding students' years of study, 188 (24.7%) were in year one, 221 (29%) were in year two, 192 (25.3%) were in year three, and 158 (21%) were in year four and above. The majority of 433 (57%) of the respondents were orthodox, followed by Protestants 230 (30.3%), Muslims 82 (10.8%), and Catholics 14 (1.9%) (Table 1).

### 3.1. Sexual Behaviour and Discussion about SRH

Three hundred twenty (42.2%) respondents said that they have a boy/girlfriend, and 224 (29.5%) have ever had sexual intercourse in their lifetime. Of those who started sexual intercourse, 178 (79.5%) stated that they had sexual intercourse within the last 12 months. More than half of the study participants (413, 54.4%) did not discuss with their parents about sexual and reproductive health issues, while 346 (45.6%) discussed. The finding is triangulated by the qualitative part as “talking about sexuality and related issues with parents or elders is not common, fearing that our parents may assume as we are engaged in forbidden activities. It is considered as rudeness if we talk about sex-related issues in front of father or mother in our home” (22-year-old male student) (Table 2).

### 3.2. Knowledge and Attitude of Students on Sexual and Reproductive Health

From a total of 759 respondents, 639 (84.2%) heard of sexual and reproductive health services. About 632 (83.3%) of the study participants were knowledgeable about sexual and reproductive health issues. Regarding the attitude towards sexual and reproductive health, 372 (49%) of the respondents had a positive attitude, while the majority of the study participants (387, 51%) had a negative attitude (Table 3).

### 3.3. Preference of Health Facilities and Sources of Information about SRH Services

Only 83 (10.9%) of study participants prefer university clinics to receive sexual and reproductive health services. A qualitative study also confirmed that the sexual and reproductive health services delivered at a university clinic do not satisfy students. “There is a contrasting pattern in reproductive health services delivery between the university clinic and private/community-based facilities. The service delivered at the university clinic has a lot of gaps, and it lacks most of the services we seek” (23-year-old female respondent). About 348 (45.8%) had received information about sexual and reproductive health from their friends. The finding from the qualitative part also showed that “…All that I know about sexual and reproductive health is from my friends or health care providers and sometimes from an advertisement on radio and TV. Based on this source, I try to understand the reproductive health services in health facilities that are available for youths like me” (21-year-old male student).

### 3.4. Utilization of Sexual and Reproductive Health Services by University Students

Of the total of 759 study participants, 378 (49.8%) utilized reproductive health services in the year before the survey. Family planning, voluntary counselling and testing of HIV, treatment for STIs, counselling on SRH, condoms, and abortion were utilized by 94 (12.4%), 289 (38.1%), 34 (4.5%), 131 (17.3), 112 (14.8%), and 11 (1.4%) of students, respectively. Among sexually active respondents, 182 (81.3%) have utilized at least one of the sexual and reproductive health services within the past twelve months.

### 3.5. Factors Associated with Sexual and Reproductive Health Service Utilization among Wolaita Sodo University Students

According to the results of the bivariate analysis, thirteen variables were associated with the utilization of sexual and reproductive health services. In the final model (multivariable analysis), the result indicated that being a first-year student, having sexual intercourse, participating in peer-to-peer discussion, and having STI syndrome were found to be significant factors associated with sexual and reproductive health service utilization (Table 4).

## 4. Discussion

This study was conducted to investigate the prevalence of sexual and reproductive health service utilization and its associated factors among Woliata Sodo university students in Ethiopia. The level of SRH service utilization was found to be 49.8%. The finding of the current study was higher than some previous studies conducted in Awobel district (41.2%) [12], Mizan Tepi University (24.5%) [13], Hadiya zone (38.5%) [14], Bahir Dar (32%) [5], Jimma [15], and Nepal (9.2%) [16]. The discrepancy in the prevalence estimate may be explained by the variation in the study settings. For example, the majority of the aforementioned studies have been conducted among secondary and high school students, whereas our study was conducted at the university which might play a role in the observed discrepancy. It is believed that university students could have a better understanding of SRH issues and such services are more accessible to them as compared to high school students. Therefore, the availability and accessibility of services may play a significant role in the observed variation in the prevalence estimate. However, the prevalence of SRH service utilization in our study was lower than the prevalence estimates from the studies conducted in Madawalabu University (80.5%) [10] and Harar town (64%) [11]. This may be due to the difference in the definition of SRH service utilization and the settings of the study. The study conducted at Madawalabu University assessed the utilization of SRH services, whereas the current study assessed utilization within one year.

In this study, being a first-year university student was associated with sexual and reproductive health service utilization. Our finding was in agreement with the findings from Madawalabu University [10]. This may be attributed to changing trends in the early development of sexual maturity and increased demand for sexual and reproductive health care [17]. Nonetheless, a study conducted in Goba town observed contrasting results [18].

Ever having sex is another factor associated with sexual and reproductive health service utilization. The current finding is in line with results from Madawalabu University [10]. Unlike the finding from the study conducted in the North Shewa Zone [19], the observation of our study was also consistent with the findings of other similar studies from Ethiopia [12, 14]. This might be due to those students who have ever had sexual intercourse needing relevant services to avoid the risk and consequences of sexual vulnerability.

Participating in peer-to-peer education is also associated with sexual and reproductive health service utilization. This finding matches with results from Madawalabu University [10], Goba town [20], Awobel district [12], and West Badewacho district [21]. The likely explanation could be that discussion about sexual and reproductive health issues helps to create awareness of the benefits of SRH services.

Having ever had STI syndrome is also another factor associated with the utilization of sexual and reproductive health services. This finding agrees with findings from a study conducted in Bahir Dar and Hadiya Zone [8, 14]. This might be due to students who had SRH problems and needed further assistance to avoid the risk and consequences of sexual vulnerability.

Among the limitations of the study, the cross-sectional nature of the study may not show the temporal relationship between the outcome and the independent variables. The current study assessed the sensitive issues related to sexuality that might have caused social desirability bias, and this may underestimate or overestimate an outcome of interest. Some of the issues raised about the sexual behaviour of participants explored the past practices of the students, which can also be affected by recall bias. Thus, the findings of this study should be interpreted with these limitations.

## 5. Conclusion

This study aims to examine sexual and reproductive health service utilization among university students and to explore the barriers to utilizing reproductive health services by university students. Even though students have good knowledge and information about sexual and reproductive health services, the utilization of their services is low, which indicates that a high level of SRH knowledge is not translating into significant behaviour change. Being a first-year student, having ever had sexual intercourse, participating in peer-to-peer discussion, and having STI syndrome were predictors of SRH service utilization. Encouraging peer support networks and establishing another integrative and comprehensive approach to improve the current reproductive health status of university students is vital.

## Figures and Tables

**Table 1:** Sociodemographic characteristics of Wolaita Sodo University students, Southern Ethiopia, 2017 (*n* = 759).

Variables	Category	Frequency	Percent
Sex	Male	411	54.2
	Female	348	45.8
Class year	I	188	24.7
	II	221	29
	III	192	25.3
	IV+	158	21
Type of school attended (at preparatory)	Governmental	613	81
	Nongovernmental	109	14
	Both	37	5
Religion	Orthodox	433	57
	Muslim	82	10.8
	Protestant	230	30.3
	Catholic	14	1.9
Marital status	Never married	721	95
	Ever married	38	5
The place for getting meal service	University cafeteria	479	63.1
	Outside university	280	36.9
Mother's occupation	Formal employment	124	16.3
	Daily labourer	8	1.2
	Self-employed/business	165	21.7
	Farmer	124	16.3
	House wife	338	44.5
Father's occupation	Formal employment	259	34.1
	Casual labourer	19	2.5
	Self-employed/business	175	23.1
	Farmer	306	40.3
Parents place of residence	Rural	412	54.3
	Urban	347	45.7

**Table 2:** Sexual behaviour and discussion about sexual and reproductive health issues among Wolaita Sodo University students, Southern Ethiopia, 2017 (*n* = 759).

Variables	Category	Frequency	Percent
Have a boyfriend/girlfriend	Yes	320	42.2
	No	439	57.8
Ever had sexual intercourse	Yes	224	29.5
	No	535	70.5
First sex consented	Yes	172	22.7
	No	52	6.8
Number of sexual partners ever had	One	106	14
	Two	57	7.5
	More than two	61	8
The last time had sex	Within the last week	33	4.3
	Within the last month	51	6.7
	Within the last six months	57	7.5
	Within the last year	37	4.9
	More than one year ago	46	6
Ever discussed with parents about SRH issues	Yes	346	45.6
	No	413	54.4
Ever participated in peer-to-peer education programs	Yes	362	47.7
	No	397	52.3
Ever had signs of STIs	Yes	47	7.2
	No	712	93.8

**Table 3:** Knowledge and attitude of Wolaita Sodo university students about sexual and reproductive health-related issues, Southern Ethiopia, 2017 (*N* = 759).

Variables	Category	Frequency	Percent
Knowledge about sexual and reproductive health issues	Knowledgeable	632	83.3
	Not knowledgeable	127	16.7
Ever heard of sexual and reproductive health services	Yes	639	84.2
	No	120	15.8
Know any family planning methods	Yes	681	89.7
	No	78	10.3
Ever heard about sexually transmitted infections	Yes	669	88.1
	No	90	11.9
Know methods for preventing STIs	Yes	596	78.5
	No	163	21.5
Attitude towards SRH service utilization	Positive attitude	372	49
	Negative attitude	387	51

**Table 4:** Bivariate and multivariable analyses showing factors associated with SRH service utilization among Wolaita Sodo University students, Southern Ethiopia, 2017.

Variables	Category	SRH service utilization		COR 95% CI	AOR 95% CI
		No	Yes		
Sex	Male	184	227	1	1
	Female	197	151	0.62 [0.46–0.83]	0.79 [0.54–1.16]
Age	17–20	187	172	0.87 [0.65–1.16]	0.81 [0.54–1.22]
	21–24	186	197	1	1
	25+	8	9	1.06 [0.40–2.81]	0.74 [0.23–2.36]
Class year	I	85	103	1.52 [1.03–2.25]	1.71 [1.10–2.69]^*∗*^
	II	123	98	1	1
	III	85	107	1.58 [1.07–2.33]	1.34 [0.85–2.13]
	IV+	88	70	0.99 (0.66–1.51]	1.06 [0.62–1.81]
Marital status	Ever married	8	30	4.02 [1.82–8.89]	1.76 [0.69–4.51]
	Never married	373	348	1	1
Place for getting meal service	University cafeteria	220	259	1	1
	Outside university	161	119	0.63 [0.47–0.85]	0.71 [0.46–1.08]
Monthly pocket money	100–500	304	296	1	1
	501–1000	72	64	0.27 [0.10–0.74]	0.79 [0.50–1.23]
	1001+	5	18	0.25 [0.09–0.70]	2.47 [0.83–7.33]
Have a boyfriend/girlfriend	Yes	131	189	1.91 [1.43–2.56]	1.29 [0.91–1.86]
	No	250	189	1	1
Ever had sexual intercourse	Yes	42	182	7.50 [5.13–10.95]	5.12 [3.33–7.90]^*∗∗∗*^
	No	339	196	1	1
Parental discussion on SRH issues	Yes	152	194	1.58 [1.19–2.12]	1.24 [0.87–1.75]
	No	229	184	1	1
Participated in peer-to-peer education	Yes	152	210	1.88 [1.41–2.51]	1.48 [1.05–2.10]^*∗*^
	No	229	168	1	1
Attitude towards SRH services	Positive	195	177	0.84 [0.63–1.12]	1.02 [0.74–1.42]
	Negative	186	201	1	1
Ever had signs of STIs	Yes	5	42	9.40 [3.68–24.04]	3.55 [1.30–9.80]^*∗*^
	No	376	336	1	1

^*∗*^ *P* < 0.05,  ^*∗∗∗*^*P* < 0.001, AOR: adjusted odds ratio, and COR: crude odds ratio.

## Data Availability

The data used to support the findings of this study are included within the article.

## Abbreviations

HIV: Human immunodeficiency virus
ICPD: International Conference on Population and Development
SRH: Sexual and reproductive health
STIs: Sexually transmitted infections.
